# London Staff Appointments

**Published:** 1914-01-31

**Authors:** 


### London Staff Appointments.

*To the Editor of* The Hospital.

Sir,—May I draw your attention to a matter connected with hospital appointments which is of great importance to many young physicians and surgeons in London just now?

As is well known, according to our present system, the would-be consultant in general medicine or surgery largely depends for his future success on gaining an appointment on the staff of a general hospital; and for many years the usual course has been for junior men to seek election to the staffs of the smaller hospitals whilst waiting for one of the vacancies—which are somewhat slow in occurring—at the various teaching hospitals.

Following this plan many have gone through life simultaneously holding appointments at both large and small hospitals. Lately, however, the demands of the leading institutions have so much increased that their authorities have felt compelled to ask the newly-appointed members of their staffs to resign whatever posts they may be holding elsewhere at the time of election.

Clearly it is not to the interest of the smaller hospitals that the younger men, however brilliant, should use them merely as stepping-stones to better work, and with the admitted intention of departing should they be successful in attaching themselves to a teaching hospital. I am informed on credible authority that in consequence of this the election committees of some of the lesser charities are pursuing an evident policy of retaliation in rejecting candidates for their vacancies who appear likely to succeed at their own hospitals.

This is, of course, a very serious thing, as the results of such a policy must inevitably be that many London men will be deprived of opportunities for obtaining the valuable clinical experience of the smaller hospitals; whilst, further, the appointments at these latter must either be left vacant or be filled by those who have scarcely achieved front-rank distinction in their year. It is said, and with apparently good reason, that this has lately happened in connection with well-known institutions.

There is a further consequence which Londoners may well take to heart—namely, that such methods of election must open the way to increased competition with provincial candidates, who naturally welcome the opportunity thus afforded of obtaining a footing in London.

Personally, I have no axe to grind, having given up my hospital appointments some time ago owing to pressure of other work, consequently I feel no diffidence in drawing your attention to this matter, which cannot be very well discussed by those on the waiting list.—Yours faithfully,

London, W. M.D. Lond.

January 21, 1914.

